# “Letter to the Editor: Treatment of infantile idiopathic scoliosis using a novel thoracolumbosacral orthosis: a case report”

**DOI:** 10.1186/s13256-022-03560-y

**Published:** 2022-12-05

**Authors:** Christian J. Fludder, Braden G. Keil

**Affiliations:** Private Practice, Melbourne, Australia

We would first like to thank and congratulate McAviney and Brown on their successful management of this case of infantile idiopathic scoliosis [1]. In a condition with little current evidence on bracing management, it is refreshing to see more current cases with positive outcomes.

We do have some points of concern. Firstly, the definition of scoliosis seen in the first sentence reads “...curvature < 10°...”. We understand this is likely a simple error as scoliosis entails a curve of > 10°. Secondly, Fig. 1c needs further clarification; previous literature, including a publication by us, lists plagiocephaly based on the side of occipital flattening [2]. This image shows rightward cervical rotation and a right occipital deformational/positional plagiocephaly not left plagiocephaly as noted on the figure [1].

**Fig. 1:**
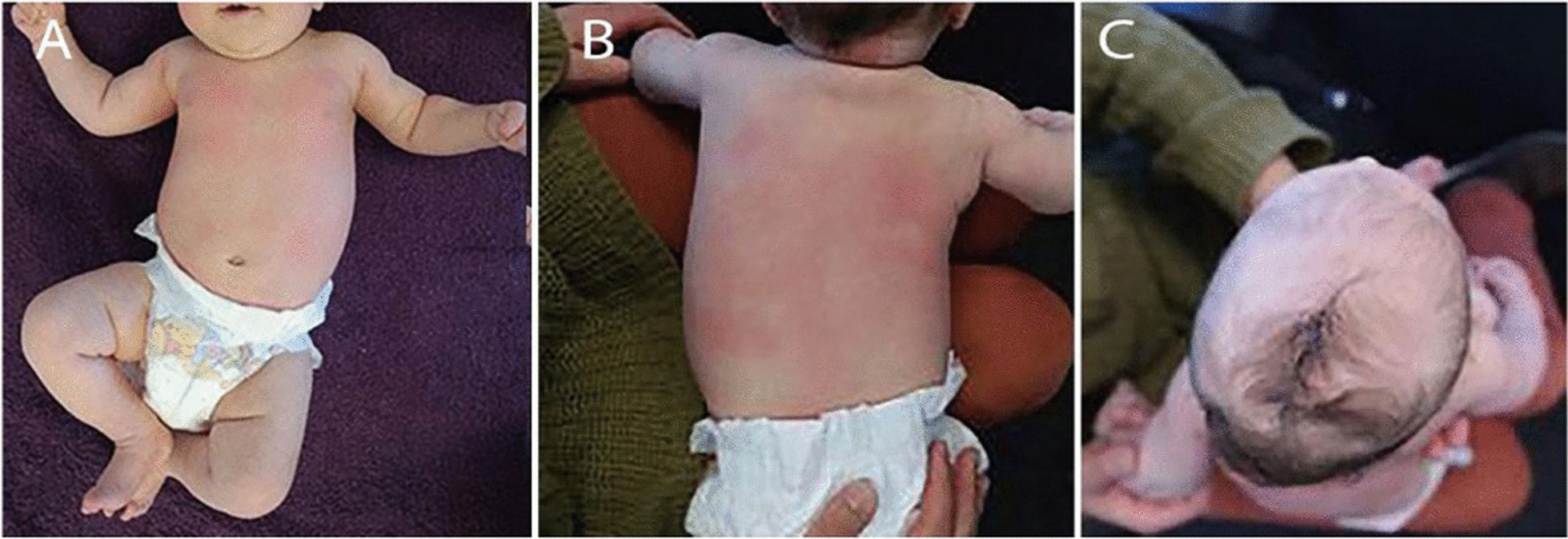
Observational findings. A scoliosis can be seen on the anteroposterior view (**a**) and posteroanterior view (**b**). Left-sided deformational plagiocephaly was observed. **c** Superior to inferior view. Figure from McAviney and Brown [1]

We also have some concerns regarding interpretation of the examination findings and management. The authors state that “The neurological examination was within normal limits except for a slight reduction in the abdominal reflexes”. Fujimori et al. (2010) makes several comments regarding infantile idiopathic scoliosis: magnetic resonance imaging (MRI) should be performed; of the neurological abnormalities that may indicate syringomyelia, an abnormal superficial abdominal reflex (SAR) may be one of the most important; and an abnormal SAR in patients with “idiopathic” scoliosis had a sensitivity of 89%, specificity of 97%, 80% positive predictive value (PPV) and 98% negative predictive value (NPV) [3]. Further to this, Lincoln (2007) also supports routine magnetic resonance imaging (MRI) of the brain in all infants with idiopathic curves of > 20°, even though spontaneous resolution is anticipated in 80–90% of cases [4]. Our concern with overlooking this neurological sign is the reported presence of neural axis abnormalities in > 20% of cases of infantile idiopathic scoliosis [5].

We also question the detail of the examination. Was there persistence of a Galant's Spinal Reflex? Unilateral persistence of the Galant's Reflex has been associated with the development of scoliosis [6]. The authors identified plagiocephaly; given the risk of developmental delay with plagiocephaly, was there any action taken to incorporate this into management? Alterations in normal cervical spine range of motion may contribute to changes of spinal tone, which in turn may be a factor in persistence of the Galant's Reflex.

A trial of manual therapy had been performed. In a study comparing physiotherapy to bracing, no significant difference was noted between the groups in terms of time to resolution, and that once resolution had commenced, no further treatment was required [7]. Previous research has reported cervical spine dysfunction in up to 94% of infants with plagiocephaly [2]. In this case was the cervical spine passive range of motion assessed by a suitably qualified pediatric chiropractor? We question the level of training of the treating practitioner, whether a different outcome may have been achieved, and whether manual therapy would have been an appropriate modality without prior MRI.

This then raises the question of whether the rate of improvement was better than that of natural history. The study that is frequently cited regarding curve resolution was performed by Mehta in 1972, which showed that 80% of all resolving curves had an initial rib-vertebral angle difference (RVAD) of < 20° and that the remaining resolving curves demonstrated a decreased RVAD on radiographs 3 months later [8]. This must be weighed against the negative side of natural progression. However in this case a curve of > 25° did put them in a higher risk category [9]. This in turn must be weighed against the accuracy of rib angle measurements, which have been demonstrated to have > 10° of error in nearly 20% of interpretations [10]. Could an error in measurement have changed the diagnosis from Progressive IIS to Resolving IIS?

We appreciate the efforts and positive outcome that was achieved by McAviney and Brown, and look forward to their response.

## Data Availability

Not applicable.
